# Ambidextrous leadership and employee voice: the mediation of benign envy and moral efficacy

**DOI:** 10.3389/fpsyg.2026.1789317

**Published:** 2026-05-15

**Authors:** Huihui Wang

**Affiliations:** Central South University, Changsha, China

**Keywords:** ambidextrous leadership, benign envy, employee voice, mediation, moral efficacy

## Abstract

This study suggests that benign envy can affect employee voice, and benign envy plays a mediating role between ambidextrous leadership and employee voice. We construct a moderated mediation model using progressive regression methods such as process. Supported by data from three time points, we examined the influence of ambidextrous leadership on employee voice. Approximately 500 samples selected different provinces and cities in different categories of domestic enterprises, one-month interval for a three-point survey. The results show that ambidextrous leadership can promote employee voice and can stimulate benign envy. Further, benign envy can mediate the effect of ambidextrous leadership on employee voice. Finally, moral efficacy can moderate the influence of ambidextrous leadership on benign envy.

## Highlights

- We construct a moderated mediation model to examine the influence.
- The study adopts a three-wave time-lagged survey design with a one-month interval between each wave, supported by data collected from participants across different provinces and cities in China.
- Approximately 500 samples in different categories of domestic enterprises was participated in the survey.

## 1 Introduction

Employee voice is an informal approach to improving the management situation and promoting the long-term development of the organization (Bandura, 1989; Lavelle et al., 2021). Voicing and reflecting on past work experiences (Li et al., 2020; Wang et al., 2022) allows the organization to stay informed about the internal situation and external changes, contributes to continuous improvement, and stimulates innovation (Tangirala and Ramanujam, 2012). Additionally, it can enhance employees’ sense of ownership and enable them to actively exert their subjective initiative. It (Morrison, 2014) is also conducive to the timely discovery and solution of problems and promotes scientific decision-making (Bain et al., 2018; House, 1996; Stamper and Masterson, 2002). Therefore, promoting effective and reasonable employee voice becomes the key to the sustainable development of the current organization. Moreover, to promote employee voice, a better understanding of its antecedents and mechanisms is required.

Scholars concur that leadership style is of great significance to employee voice (Sherf et al., 2021). Previous studies have delved into the influence of single leadership styles on voice, such as authentic leadership, transformational leadership, and ethical leadership (Detert and Burris, 2007). Nevertheless, these studies have not taken into account the impact of “both-and” leadership. Ambidextrous leadership is a form of leadership style that consists of two distinct yet complementary leadership behaviors, representing the extension and application of ambidextrous leadership theory in the leadership domain (Pinae Cunha et al., 2019). In comparison to a single leadership style, ambidextrous leadership caters to the requirements of organizational competition and sustainable development in complex and dynamic environments (Mascareño et al., 2021). Consequently, ambidextrous leadership is emerging as the most prevalent leadership style in organizations. Thus, does this type of leadership have the same effect on employee voice as a single leadership style? How does it impact employee voice?

Based on the theory of emotion as social information (EASI), this paper discusses whether and how ambidextrous leadership influences employee voice. According to EASI, emotion is a type of social information that reflects individual cognition and attitude, and expressing emotion has a signaling function in interpersonal decision-making (Van Dyne and LePine, 1998). Emotion is the most genuine psychological experience of employees prior to action. Leadership behavior is the most direct and crucial factor affecting employees’ emotions (Warrier et al., 2022; Ayoko, 2023). Moreover, emotions further influence behavior in more competitive settings, and benign envy is one such emotion. According to the EASI model, leadership style may affect benign envy, which in turn affects employee behavior, such as voice behavior. Therefore, based on the EASI model, this study explores the role of benign envy as a mediating mechanism between ambidextrous leadership and employee voice.

Moral efficacy is the belief “in one’s capabilities to organize and execute the courses of action required to produce given levels of attainments.” It affects thoughts, feelings, and behavior (Paciello et al., 2023; Fisk and Friesen, 2012). Based on this, we select moral efficacy as the moderator in this study.

**Figure 1:**
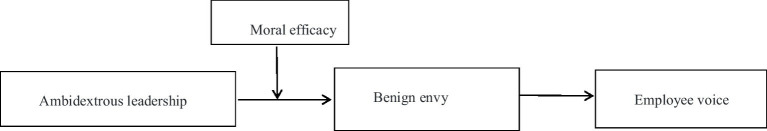
Research model.

This study builds a moderated mediation model (Figure 1) to explore whether and how ambidextrous leadership influences employee voice. This study makes three contributions:

First, by discussing the influence of ambidextrous leadership on employee voice, this study extends the research on the antecedents of employee voice from single leadership to “both-and” leadership styles. Second, based on the EASI model, this study attempts to explain the mechanism between ambidextrous leadership and voice by establishing a mediated mechanism. Moreover, it reveals the black box of the relationship between ambidextrous leadership and employee voice, expanding the intention and application scope of the EASI model. Third, by discussing the moderating effect of moral efficacy between ambidextrous leadership and benign envy, this study expands the boundary condition of the relationship between ambidextrous leadership and benign envy. As such, this paper constructs a more perfect model to research the relationship between ambidextrous leadership and employee voice.

## 2 Theoretical overview and hypotheses development

The study has utilized the EASI model as a backdrop to explain, inform, and gain a better understanding of the hypothesized relationships proposed in the study model.

### 2.1 Ambidextrous leadership and employee voice

The voice refers to “when employees become discontented with the organizational status quo, they voice their dissent through protests or petitions, striving to reform the existing conditions rather than hastily abandoning their posts, all to steer the institution back toward its proper developmental course.” Liang et al. (2019) emphasizes that employee voice is passively initiated by employees dissatisfied with organizational environment changes (Liang et al., 2012). Moreover, Van Dyne and LePine (1998) suggests that employees’ voices are a kind of interpersonal communication behavior in the organization (Van Dyne and LePine, 1998). As such, they will offer constructive suggestions to change the organizational status and improve organizational performance, making it more creative (Van Dyne and LePine, 1998).

This study posits that ambidextrous leadership has a positive influence on employee voice. Ambidextrous leadership empowers employees by delegating decision-making authority and holding them accountable for their work actions. This fosters a strong sense of responsibility among employees, which in turn facilitates them in presenting their views and ideas (Zheng et al., 2017).

Moreover, ambidextrous leadership adheres strictly to organizational rules and procedures. It carefully guides employees’ behavior and clarifies the purpose and significance of the work to address potential (Mascareño et al., 2021) issues.

In this context, employees will take charge of their own development, and enterprise goals will be closely correlated with organizational values. When employees are in alignment with organizational values, they are more inclined to contribute to the organization through out-of-role behaviors such as voicing their opinions.

In summary, we expect that:

*Hypothesis 1*: Ambidextrous leadership is positively related to employee voice.

### 2.2 Benign envy as mediator

Benign envy consists of desire, enhanced motivation, and emulation (Demirtas et al., 2017). Recently, studies have shown that leadership can evoke benign envy (Pan et al., 2021). Notably, leadership is the most direct and crucial factor influencing employee benign envy (Andiappan and Dufour, 2020). Ambidextrous leadership, which takes both aspects into account, will, to some extent, pay more attention to employee emotions (Festinger, 1954; Luo et al., 2018). Moreover, ambidextrous leadership creates a relaxed environment (Mascareño et al., 2021) where employees feel respected. Consequently, this triggers benign envy, motivates employees, and boosts work performance. Thus, ambidextrous leadership can foster the benign envy of employees.

Benign envy can also drive voice (Lange and Weidman, 2018; Thiel et al., 2021). An employee’s ability to effectively implement voice depends partly on their ability to understand and express their envy (Shaffakat et al., 2022). Benign envy is characterized by feelings of liking and admiration for the envied, as well as an increased motivation to excel. This aligns benign envy with action behaviors oriented toward raising one’s own level to that of the envied target (Odle, 2014).

Benign envy can also stimulate employees’ motivation (Pan et al., 2021) and help promote the formation of employees’ sense of ownership, enabling them to provide voice. According to the previous argument, ambidextrous leadership can trigger benign envy, and in turn, benign envy can promote voice. Thus, this study suggests that benign envy may mediate the direct relationship between ambidextrous leadership and voice. Based on individual-level research, an employee’s individual psychological emotions will lead to the generation of voice behavior (Duan et al., 2023). Consequently, we hypothesize that:

*Hypothesis 2*: Benign envy plays a positive mediating role in the influence of ambidextrous leadership on employee voice.

### 2.3 Moral efficacy as a moderator

Moral efficacy is the primary proximal cognitive variable that affects behavior (Bandura, 1989; Shepherd et al., 2021). It enables individuals to reflect on their actions. People with moral efficacy can monitor themselves and their actions (Frommer et al., 2021). Different levels of moral efficacy also influence their behavior (Paciello et al., 2023).

A high level of moral efficacy means that employees can establish rules to ensure the success of their efforts (Tang and Wei, 2021). Individuals with low moral efficacy are more likely to hesitate at a suggestion because they fear it will go wrong (Bandura, 1989; Podsakoff et al., 1990). Moreover, moral efficacy can moderate the relationship between ambidextrous leadership and benign envy (high moral efficacy leads to benign envy). Thus, this study hypothesizes that moral efficacy can moderate the relationship between ambidextrous leadership and benign envy. In summary, we expect that:

*Hypothesis 3*: Moral efficacy positively moderates the positive relationship between ambidextrous leadership and benign envy. The higher an individual’s moral efficacy, the stronger the relationship between ambidextrous leadership and benign envy; conversely, the lower an individual’s moral efficacy, the weaker this relationship.

*Hypothesis 4*: Moral efficacy positively moderates the mediating effect of benign envy on the relationship between ambidextrous leadership and employee voice. That is, the higher the moral efficacy, the stronger the mediating effect of benign envy on the relationship between ambidextrous leadership and employee voice; conversely, the mediating effect is weaker.

## 3 Methodology

### 3.1 Measures

The measures were all adapted from the literature. The study employed these scales because they all had reliable and valid reports from prior studies. All survey items were measured on a 5-point Likert scale, ranging from 1 to 5, and loaded highly on their latent construct.

*Ambidextrous leadership*: We measured ambidextrous leadership at time 1, using the scale developed by Zacher and Rosing (2012), which comprises 14 items, such as “Encourages experimentation with different ideas” and “Controls adherence to rules.” Cronbach’s alpha was 0.85.

*Control variables*: In addition, we measured control variables at time 1. We followed the recommendations of Spector and Brannick (2011) to control for variables that might influence the hypothesis. Several empirical studies suggest that gender, age, education, tenure, and position are related to assessing one’s tendency to give voice (Liang et al., 2012; Park et al., 2017). Research indicates that employees in higher organizational positions may feel more obligated to engage in voice (Fuller et al., 2006). Education level was measured with three categories: junior college and below, undergraduate, and master’s degree or above. Furthermore, organizational tenure was measured as the number of months worked in the company. Position in the organization was measured with three categories: employees, middle managers, and senior managers. Working years were measured with four categories: less than 1 year, 1–3 years, 3–5 years, and more than 5 years.

*Moral efficacy*: We used the scale developed by Ogunfowora et al. (2021), which is a 3-item scale to test moral efficacy at time 2. A sample item is “I am confident that I can confront others who behave unethically to resolve the issue.” Cronbach’s alpha was 0.82.

*Benign envy*: Benign envy was measured at time 2, using 4 items adapted from Lange and Weidman (2018), such as “I wanted to work harder to also obtain exactly X.” Cronbach’s alpha was 0.82.

*Employee voice*: We measured employee voice at time 3 once again. We also measured employee voice at time 1, using the scale developed by Liang et al. (2012), which comprises 10 items, such as “Proactively voice out constructive suggestions that help the unit reach its goals.” Cronbach’s alpha was 0.85.

#### 3.1.1 Sample and data collection

Following the rules of the data survey, we selected a portion of the data from the western provinces of China. We assured respondents of the confidentiality and anonymity of the data. Our sample is from four companies in western China, covering the IT, manufacturing, mining, and comprehensive industries.

Before the formal survey, we interviewed the human-resource managers and executives of these companies. To reduce common method bias in the surveys, we sent out surveys at three time points, each separated by 1 month. At the first time point, we measured ambidextrous leadership and control variables; 408 participants completed the first survey.

A month later, at the second time point, we measured benign envy and moral efficacy. In total, 352 participants completed the survey at the second time point, with a response rate of 86.30%.

Finally, 1 month after the second time point, we measured employee voice; 286 participants completed all three time-point surveys, with a final response rate of 70.10%.

In terms of demographics, 56.80% of the participants were male. Their average age was 37.90 (SD = 9.39 years), ranging from 22 to 59 years. Their average organizational tenure was 3.90 years (SD = 2.33 years). Regarding educational attainment, 24.50% had received higher education at the undergraduate level or above. Furthermore, those with 1–3 years of work experience accounted for 54.50%, and those with more than 4 years of work experience accounted for 45.50%.

#### 3.1.2 Measurement model analysis

To test the construct validity of the measurement tools for each research variable, this study adopted Confirmatory Factor Analysis (CFA) to fit and test the measurement models of the four core variables: ambidextrous leadership, benign envy, moral efficacy, and employee voice. AMOS 26.0 software was used, and Maximum Likelihood Estimation (MLE) was employed for model estimation.

The model was set as follows: A confirmatory factor model containing four latent variables (ambidextrous leadership, benign envy, moral efficacy, and employee voice) was constructed, with each latent variable corresponding to its measurement items. Each item only loaded on its corresponding latent variable without cross-factor loading, ensuring the independence and rationality of the measurement model.

The model fitting results showed that all fitting indices met the commonly accepted academic standards: Comparative Fit Index (CFI) = 0.923, Tucker-Lewis Index (TLI) = 0.918, both greater than 0.90; Root Mean Square Error of Approximation (RMSEA) = 0.057, with a 95% confidence interval of [0.051, 0.063], less than 0.08; Standardized Root Mean Square Residual (SRMR) = 0.042, less than 0.08.

The above results indicate that the measurement model fits well, and the construct validity of the measurement tools for each variable is high, which can be used for subsequent research analysis.

### 3.2 Common method bias test

All data in this study were collected through questionnaire surveys. To avoid the impact of common method bias on the research results, Harman’s single-factor test was used. SPSS was used to conduct an unrotated exploratory factor analysis on all measurement items. The results showed that a total of 4 common factors with eigenvalues greater than 1 were extracted, accounting for 61.928% of the cumulative explained variance, among which the explained variance of the largest factor was 37.21%, lower than the critical value of 40%. This indicates that there is no serious common method bias in this study, which will not have a substantial impact on the research conclusions, and the research results are reliable.

## 4 Data analysis and results

### 4.1 The direct effect between ambidextrous leadership and employee voice

Descriptive statistics and correlations are presented in Table 1, which shows the mean, standard deviation, and correlation coefficients of the variables. The table indicates that ambidextrous leadership was positively correlated with employee voice (*r* = 0.19, *p* < < 0.05), thus supporting Hypothesis 1. Additionally, ambidextrous leadership was positively associated with benign envy (*r* = 0.21, *p* < 0.05), and benign envy was associated with employee voice (*r* = 0.16, *p* < 0.05). To test the potential multicollinearity between variables, this study used the Variance Inflation Factor (VIF) for testing. The results showed that the VIF values of all variables ranged from 1.23 to 2.78. Among them, the VIF values of core variables were as follows: ambidextrous leadership 1.89, benign envy 2.12, moral efficacy 2.78, and employee voice 2.05; the VIF values of control variables were: gender 1.23, age 1.45. All VIF values were far below the critical value of 10 and less than 5, indicating that there was no serious multicollinearity problem in this study. The model setting was reasonable and stable, and the research results were reliable.

**Table 1:** Descriptive statistics and correlations.

Variables	Mean	SD	1	2	3	4	5	6	7
1. Gender	1.25	0.43							
2. Age	36.17	9.05	−0.17^*^						
3. Education	1.17	0.39	−0.04	−0.22^**^					
4. Occupation	1.14	0.38	−0.13	0.01	0.41^**^				
5. Ambidextrous leadership	5.03	0.82	−0.06	−0.02	0.17^*^	0.18^*^			
6. Employee voice	4.89	1.61	0.15^*^	−0.26^**^	0.19^**^	0.16^*^	0.19^**^		
7. Moral efficacy	5.11	1.06	−0.08	0.02	0.08	0.18^**^	0.62^**^	0.17^*^	
8. Benign envy	4.93	1.26	−0.04	−0.05	−0.01	0.06	0.20^**^	0.21^**^	0.19^**^

^*^, ^**^, ^***^ denote *p* < 0.05, *p* < 0.01, *p* < 0.001, respectively. Gender: 1 = male, 2 = female; education: 1 = junior college and below, 2 = junior college, 3 = undergraduate, 4 = master and above; working life: 1 = less than 1 year, 2 = 1–3 years, 3 = 3–5 years, 4 = more than 5 years; Management level: 1 = general staff, 2 = middle management, 3 = senior management.

### 4.2 The mediating effect test of benign envy

The mediating effect of benign envy was tested using the bootstrap sampling method (95% confidence interval) and progressive regression. Table 2 presents the results regarding the role of benign envy in the relationship between ambidextrous leadership and employee voice.

**Table 2:** Main effect and mediating effect analysis results.

Variables	Benign envy				Employee voice					
	Model 1		Model 2		Model 3		Model 4		Model 5	
	*B*	SE	*B*	SE	*B*	SE	*B*	SE	*B*	SE
Control variables										
Gender	−0.08^*^	0.21	−0.06^**^	0.20	−0.02^**^	0.21	0.63^**^	0.25	0.67^*^	0.25
Education	−0.13^*^	0.25	−0.21^*^	0.25	−0.26^*^	0.25	0.59^**^	0.31	0.52^*^	0.31
Position	0.26^*^	0.26	0.18^*^	0.26	0.21^*^	0.26	0.59^**^	0.32	0.61^*^	0.32
Independent variables										
Ambidextrous leadership			0.31^***^	0.11	0.38^*^	0.11	0.30^**^	0.13	0.23^***^	0.14
Mediated variables										
Benign envy					0.15^*^	0.08			0.24^*^	0.09
*R* ^2^	0.01^**^		0.05^*^		0.07^**^		0.10^*^		0.13^*^	
Δ*R*^2^	0.01^**^		0.04^*^		0.07^**^		0.02^**^		0.03^*^	
*F*	0.42^***^		2.41^***^		2.71^***^		5.13^***^		7.50^***^	
Mediated effect	effect		BootSE		BootLLCI	BootULCI				
Benign envy	0.04		0.02		0.01	0.08				

*p* < 0.05, **p < 0.01, ***p < 0.001.

The results showed that the effect of ambidextrous leadership on benign envy was 0.04. Additionally, the indirect effect of ambidextrous leadership on employee voice via benign envy was 0.04 (*p* < 0.01), with a 95% confidence interval (CI) of [0.0054, 0.0773], excluding 0.

Thus, these findings suggest that benign envy mediates the relationship between ambidextrous leadership and employee voice, and Hypothesis 2 is supported.

### 4.3 The moderating effect of moral efficacy

**Figure 2:**
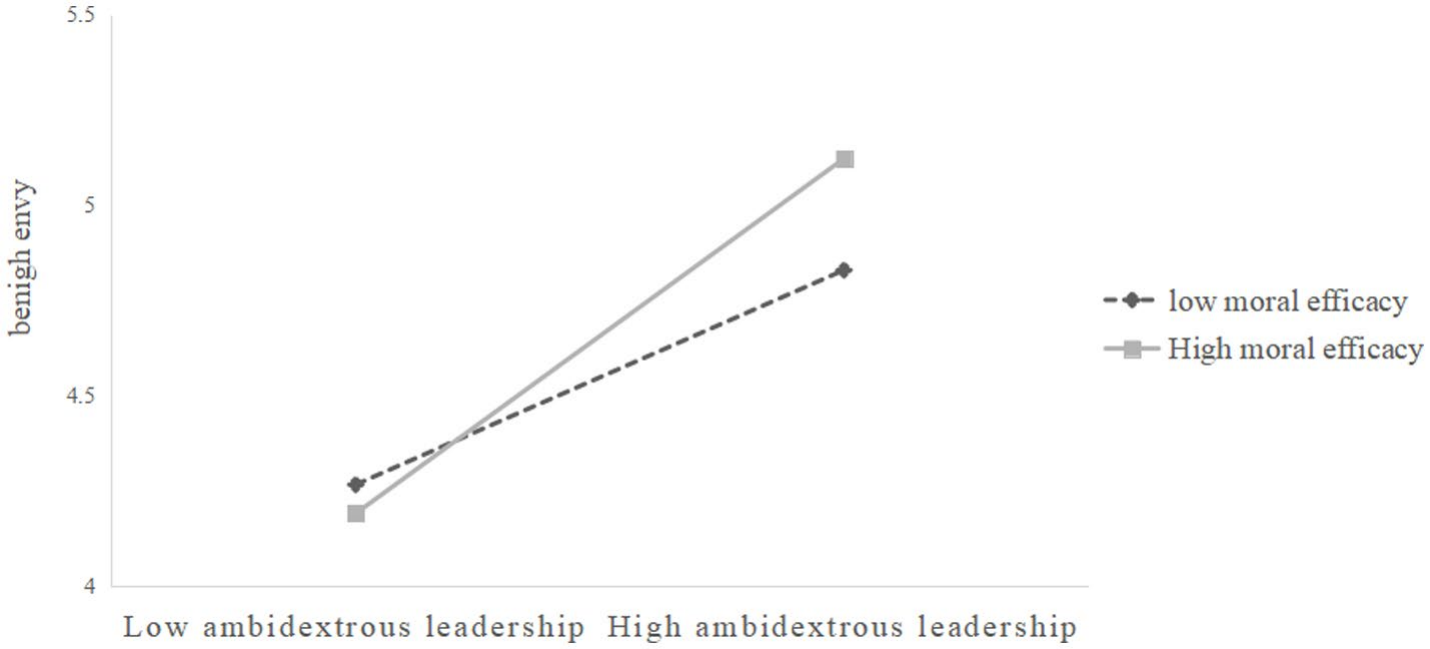
Regulatory effect diagram.

To test the moderating effect, the control variables, ambidextrous leadership, moral efficacy, and their interaction terms were entered into the regression equation, with benign envy as the dependent variable. The moderating effect of moral efficacy on the relationship between ambidextrous leadership and benign envy is shown in Table 3. The effect value was 0.08 (*p* < 0.01), with a 95% (CI) [0.01, 0.33], not including 0. The moderated effect existed. This study drew the moderation effect figure of moral efficacy and conducted a gradient analysis, as shown in Figure 2. The results showed that the higher the moral efficacy, the stronger the positive effect of ambidextrous leadership on benign envy. Conversely, when the effect is weaker, then Hypothesis 3 is supported.

**Table 3:** Analysis results of moderating of moral efficacy.

Variables	Benign envy			
	*B*	SE	LLCI	ULCI
Control variables				
Gender	−0.01^**^	0.20	−0.41	0.39
Education	−0.25^**^	0.25	−0.74	0.24
Position	0.18^**^	0.26	−0.33	0.68
Independent variables				
Ambidextrous leadership	0.28^**^	0.14	0.02	0.55
Moderated variables				
Moral efficacy	0.11^**^	0.11	−0.10	0.32
Interactive item				
Ambidextrous leadership * moral efficacy	0.17^**^	0.08	0.01	0.33

**p < 0.01.

### 4.4 Moderated mediate effect

Hypothesis 4 predicted that the relationship between ambidextrous leadership and employee voice through benign envy would be moderated by moral efficacy. The bootstrap method was employed to test the moderated mediation effect, and the results are presented in Table 4.

**Table 4:** Results of moderated mediation effect analysis.

Moderator	Ambidextrous leadership		Benign envy	Employee voice	
	Phase		Effect		
	Phase I	Phase II	Direct effect	Indirect effect	Total effect
Low score group	0.10	0.07	0.23^**^	0.07^**^	0.30^**^
High score group	0.46	0.11			
Between-group differences		0.04			

**p < 0.01.

Table 4 shows that the effect analysis of employee voice indicates moderated mediating effects. As depicted in Table 4, the difference between these indirect effects was significant (diff = 0.04). Therefore, moral efficacy moderates the relationship between ambidextrous leadership and employee voice through benign envy. Thus, Hypothesis 4 is supported.

## 5 Discussion

The importance of voice in organizational development has been discussed in numerous studies (Ng et al., 2021; Zheng et al., 2021), and the impact of leadership on voice has also been investigated (Sherf et al., 2021). However, the mechanism through which ambidextrous leadership affects voice has not been thoroughly explored.

This study analyzed the mediating effect of benign envy and the moderating effect of moral efficacy to uncover the mechanism between ambidextrous leadership and voice. Confirmation of the mediating role of benign envy in the context of moderated moral efficacy identified the boundary conditions for the relationship between ambidextrous leadership and voice.

Taken together, the results of a series of research reveal the following: First, ambidextrous leadership promotes employee voice. Second, benign envy plays a mediating role in the influence of ambidextrous leadership on employee voice. Finally, moral efficacy can moderate the relationship between ambidextrous leadership and employee voice through benign envy.

### 5.1 Theoretical implications

The results of this study make several significant contributions.

First, this study expands the research on the relationship between leadership style and employee voice from a single leadership style to a “both-and” leadership style. It discovers that ambidextrous leadership can promote employee voice (Rosing et al., 2011). This conclusion aligns with previous studies. It broadens the research on the outcome variables of ambidextrous leadership and the antecedent variables of employee voice. Moreover, the study establishes the direct relationship between ambidextrous leadership and employee voice, demonstrating a significant positive correlation between them.

Second, by establishing connections, this study explains the mediating mechanisms through which ambidextrous leadership is positively related to benign envy. Based on the EASI theory, this study unlocks the “Black Box” of the role of ambidextrous leadership in employee voice. Notably, it reveals that ambidextrous leadership indirectly affects employee voice through benign envy. It also enhances the mediating mechanism between ambidextrous leadership and employee voice. Moreover, it indicates that benign envy is a new mediating mechanism linking ambidextrous leadership and employee voice, enriching the research on mediation variables between them. By establishing a mediating mechanism through benign envy, it overcomes the limitations of previous studies (Hu et al., 2020; Rosing et al., 2011).

Finally, consistent with previous studies, we found that there are boundary conditions between ambidextrous leadership and employee voice. However, existing research has not yet explored the boundary conditions of the ethical efficacy of ambidextrous leadership on employee voice. Thus, this study expands the boundary conditions and makes a useful addition to the research on subordinate interaction. It does so by identifying the positive moderating effect of moral self-efficacy on the relationship between ambidextrous leadership and benign envy, and by establishing the positive moderating effect of ambidextrous leadership on employee voice through employee benign envy.

### 5.2 Practical implications

First, managers should pay attention to their leadership style, such as ambidextrous leadership, to effectively promote employee voice. Additionally, managers should be aware of the importance of employee voice to the organization’s sustainable development, as leadership can affect employee voice. Therefore, managers must encourage employee voice and promote the influence of ambidextrous leadership on voice through benign envy and moral efficacy.

Second, employees should understand the role of benign envy and ethical efficacy, as they can drive employee voice. Therefore, employees should pay attention to the generation of benign envy and ethical efficacy. Thus, in the actual work of enterprises, employees should pay attention to the vital role of moral efficacy.

## 6 Limitations and future research directions

First, we only included gender, position, and years, which were selected as control variables. Future research may need to expand on more aspects of ambidextrous leadership as a control variable to study the impact of employee voice. Second, we have only examined benign envy as a mediated mechanism from the perspective of an emotional mechanism, and there may be other mediated mechanisms. Thus, future research should explore other mediated mechanisms to expand the existing research.

## 7 Conclusion

This study shows that ambidextrous leadership promotes employee voice, and benign envy plays a mediated role in the role of ambidextrous leadership on employee voice. These findings suggest managers should be aware of stimulating their internal voice needs. To a certain extent, leaders can stimulate employee benign envy and moral efficacy and promote employee voice. Ultimately, they can promote the sustainable development of enterprises.

## Data Availability

The original contributions presented in the study are included in the article/supplementary material, further inquiries can be directed to the corresponding author.
